# Automated code generation from LEMS, the general purpose model specification language underpinning NeuroML2

**DOI:** 10.1186/1471-2202-15-S1-P45

**Published:** 2014-07-21

**Authors:** Boris Marin, Padraig Gleeson, Matteo Cantarelli, Robert Charles Cannon, Robin Angus Silver

**Affiliations:** 1 Department of Neuroscience, Physiology and Physiology, University College London, London, UK; 2 CAPES Foundation, Ministry of Education of Brazil, Brasilia, DF, Brazil; 3 Metacell LLC, San Diego, California, USA; 4 Textensor Limited, Edinburgh, UK

## 

Making models more transparent, reproducible and accessible is a major challenge in computational neuroscience. The wide range of modelling tools and strategies available, while convenient from a pragmatic point of view, often make model reuse harder. Efforts on standardization [1], model sharing [2,3] and practices learned from the open-source community [3-5], on the other hand, are proving increasingly invaluable in improving the transparency and reusability of computational models. NeuroML2, the latest version of a model description language for computational neuroscience, is built on LEMS (Low Entropy Model Specification, [6]) a general purpose model specification language. LEMS has a highly structured, hierarchical format, rooted in physical principles, which allows the definition of physical systems including arbitrary networks of nodes modeled as hybrid dynamical systems. There are currently two LEMS interpreters, jLEMS [5] and PyLEMS [6], implemented in Java and Python respectively. As reference implementations, numerical performance and accuracy are not their primary goals. In order to simulate NeuroML2/LEMS models in an accurate, robust and efficient way it is important to be able to perform the numerical integration using well tested scientific simulators, both neuroscience specific, e.g. *NEURON*, and general purpose, e.g. *XPPAUT*, *MATLAB*. Such simulators provide a robust numerical infrastructure important for modelling in general, such as high-order ODE integration and accurate event detection.

We have developed a set of template-based code generators for LEMS whose goal is to transform a LEMS model into a number of representations compatible with different simulators, using back-end specific templates to produce native code. The translation of the models happens through an intermediate format, developed for this purpose, named dLEMS (distilled LEMS). The dLEMS abstraction is ODE centric for convenience of interoperability with the different target languages. It represents a flattened (e.g. hierarchical channel descriptions are reduced to the core ODEs) and dimensionless version of the LEMS model. The advantage of this approach, compared for instance to a compiler based strategy, is that users willing to provide support for a target simulator can more easily work with dLEMS, as it closely reflects the ODE representations used in general purpose simulators. Users will simply have to create new templates for their target simulator or modify existing ones. Our initial results show that the template-based code-generation approach is feasible, producing runnable simulations in a variety of platforms for single cell models – in particular, templates are available for the following platforms: *C* code generation (using the LLNL Sundials library for numerical integration), *XPPAUT*, *MATLAB* and *Modelica*. All tools we have developed for this are open source and freely available. We are presently working at extending the dLEMS specification of connections between elements, creating a simplified format for template based mapping to neuronal simulators.
